# Acquired TPM3::NTRK1 fusion as a novel off-target resistance mechanism to selpercatinib in RET-positive lung adenocarcinoma: a case report

**DOI:** 10.3389/fonc.2026.1936583

**Published:** 2026-08-28

**Authors:** Ritwika Choudhury, Mansi Sharma, Abhinav Dewan, Sabeena K. Choudhary, Priyanka Venkatraman, Vivek Subbiah, Ullas Batra

**Affiliations:** 1 Department of Research (Medical Oncology Unit 5), Rajiv Gandhi Cancer Institute & Research Centre, New Delhi, India; 2 Department of Medical Oncology, Rajiv Gandhi Cancer Institute & Research Centre, New Delhi, India; 3 Stanford Cancer Institute, Division of Oncology, Stanford University School of Medicine, Palo Alto, CA, United States

**Keywords:** case report, novel mutation, NSCLC - lung adenocarcinoma - RET, NTRK fusion, RET mutation, selpercatinib resistance

## Abstract

Acquired resistance remains a major challenge in RET fusion-positive non-small cell lung cancer treated with selective RET inhibitors. NTRK fusions represent a rare off-target resistance mechanism in this setting, with limited clinical data to guide management. We report the first case of an acquired TPM3::NTRK1 fusion as a resistance mechanism to selpercatinib in KIF5B::RET-positive lung adenocarcinoma. A male in his late 40s, a non-smoker, underwent surgical resection for NSCLC after incidental detection of a pulmonary lesion. Following disease recurrence, KIF5B::RET was identified as the sole oncogenic driver and the patient achieved durable disease control for 46 months on selpercatinib, with three episodes of oligoprogression managed by stereotactic body radiotherapy. At systemic progression, RNA-inclusive tissue next-generation sequencing (Guardant360 Tissue) identified TPM3::NTRK1 (variant allele fraction 30.9%; RNA 6,109 transcripts per million) and residual KIF5B::RET (variant allele fraction 6.9%), with no secondary RET kinase domain mutations. A concurrent liquid biopsy (cfDNA) assay failed to detect either fusion, with tumor fraction <0.05%, highlighting the limitations of cfDNA-based platforms for fusion detection at low tumor shedding. Combination selpercatinib and larotrectinib was commenced, demonstrating metabolic response on PET-CT at eight weeks with concurrent clinical improvement. This case identifies acquired NTRK1 fusion as a rare, actionable bypass resistance mechanism to selective RET inhibition, distinct from previously reported NTRK3 fusions, and underscores the importance of RNA-based tissue reassessment at progression to guide rational combination targeted therapies.

## Introduction

RET fusions define a distinct molecular subset of non-small cell lung cancer (NSCLC), and with the advent of selective RET inhibitors like selpercatinib, the therapeutic landscape has undergone a paradigm shift. Despite durable responses, with updated median progression-free survival of 22 months (3), acquired resistance remains an anticipated consequence of sustained targeted therapy. While off-target bypass mechanisms involving acquired NTRK fusions have been reported following selective RET inhibition (6) (8), NTRK1-mediated resistance has not previously been described. Here, we report an acquired TPM3::NTRK1 fusion arising as a bypass resistance mechanism to selpercatinib in KIF5B::RET-positive lung adenocarcinoma, demonstrating a clinically meaningful radiological response to concurrent RET and TRK inhibition guided by real-time genomic profiling.

## Case presentation

A male in his late 40s, a non-smoker, presented with a 13x15 mm left upper lobe pulmonary nodule. Biopsy confirmed lung adenocarcinoma, and PET-CT demonstrated localized disease. Left upper lobectomy confirmed pT1aN0 moderately differentiated adenocarcinoma and no adjuvant therapy was given. Disease recurrence was confirmed after 16 months using a CE-IVD 52-gene next-generation sequencing panel covering both DNA and RNA workflows on FFPE tissue, including hotspots, SNVs, indels, CNVs, and gene fusions which revealed a KIF5B::RET fusion as the sole oncogenic driver (EGFR wild-type, ALK, ROS1, MET, and PD-L1 negative).

At the time of KIF5B::RET identification, selpercatinib was not commercially available in India and financial constraints precluded immediate access. The patient was therefore treated with six cycles of pemetrexed-cisplatin followed by prolonged pemetrexed maintenance (~54 cycles). Repeat NGS at progression reconfirmed KIF5B::RET as the sole driver. Nab-paclitaxel bridging (5 cycles) preceded selpercatinib initiation via compassionate access at Month 60 (**Table 1**).

**Table 1:** Disease course and key interventions.

Date	Event
Month 1	Left upper lobectomy + mediastinal node dissection. HPE: pT1aN0 moderately differentiated adenocarcinoma. No adjuvant therapy.
Months 16-20	Recurrence confirmed. NGS: KIF5B::RET (sole driver). Pemetrexed+ cisplatin x 6 ---> partial response. Pemetrexed maintenance commenced.
Months 21-59	Pemetrexed maintenance (~54 cycles). At progression: repeat biopsy and NGS reconfirmed KIF5B::RET Months 58-59: nab-paclitaxel x 5 (bridge) ---> stable disease.
Month 60	Selpercatinib commenced.
Month 80	Oligoprogression: lymph node metastases (new right internal mammary and anterior phrenic nodes). Biopsy: metastatic adenocarcinoma. SBRT 35 Gy/5# and Selpercatinib continued.
Months 91-92	Oligoprogression: increase in size of left lower lobe nodule. Biopsy: residual adenocarcinoma. SBRT 45 Gy/3# and Selpercatinib continued.
Months 99-102	New sternal lytic lesion. SBRT 35 Gy/5#. Month 102 PET-CT: sternal lesion regressing, otherwise stable.
Month 105	Systemic progression. Hyponatraemia (Na^+^ 103 mmol/L). Increase in bilateral pulmonary disease, loculated left pleural effusion, increased right pleural effusion (L>R) and new pleural involvement [**Figure 1**] Left pleural effusion-IPC inserted. Weekly paclitaxel as bridge. CT-guided biopsy (right lung nodule)+ Guardant360 Tissue NGS + Guardant360 Liquid (cfDNA) assay (blood)
Month 106	NGS: TPM3::NTRK1 (VAF 30.9%; RNA 6,109 TPM) + KIF5B::RET (VAF 6.9%; RNA 2,942 TPM). [**Figure 2**] No secondary RET mutations. Co-alterations: CDKN2A homozygous deletion, ATM/CHEK2/BRCA2/RB1 loss, MTAP loss, RAD51C methylation; TMB 7.97 mut/Mb; MSS, PD-L1 <1%. Guardant360 Liquid (cfDNA) assay: No somatic alterations
Month 106	Selpercatinib 120--->160 mg BD + larotrectinib 100 mg BD commenced
Month 107	4-week review: good tolerability; Na^+^ 116 mmol/L Patient- ambulatory IPC draining 200-250 mL/3-4 days.
Month 108	8-week PET-CT: metabolic response, lesion size reduced, predominantly consolidative change. [**Figure 2**] No adverse drug reactions. Na^+^ 128 mmol/L

The patient maintained disease control on selpercatinib for 46 months. Three episodes of oligoprogression: nodal (new right internal mammary and anterior phrenic lymph nodes), pulmonary (increasing left lower lobe nodule), and skeletal (sternal lytic lesion) were managed with SBRT while selpercatinib was continued. Serial brain MRI showed no intracranial metastases (**Table 1**).

At Month 105, PET-CT demonstrated systemic progression with increasing bilateral pulmonary disease and bilateral pleural effusions. Clinically, the patient developed dyspnoea and severe hyponatraemia secondary to SIADH. An indwelling pleural catheter was inserted and weekly paclitaxel was initiated while molecular profiling was undertaken.

**Figure 1:**
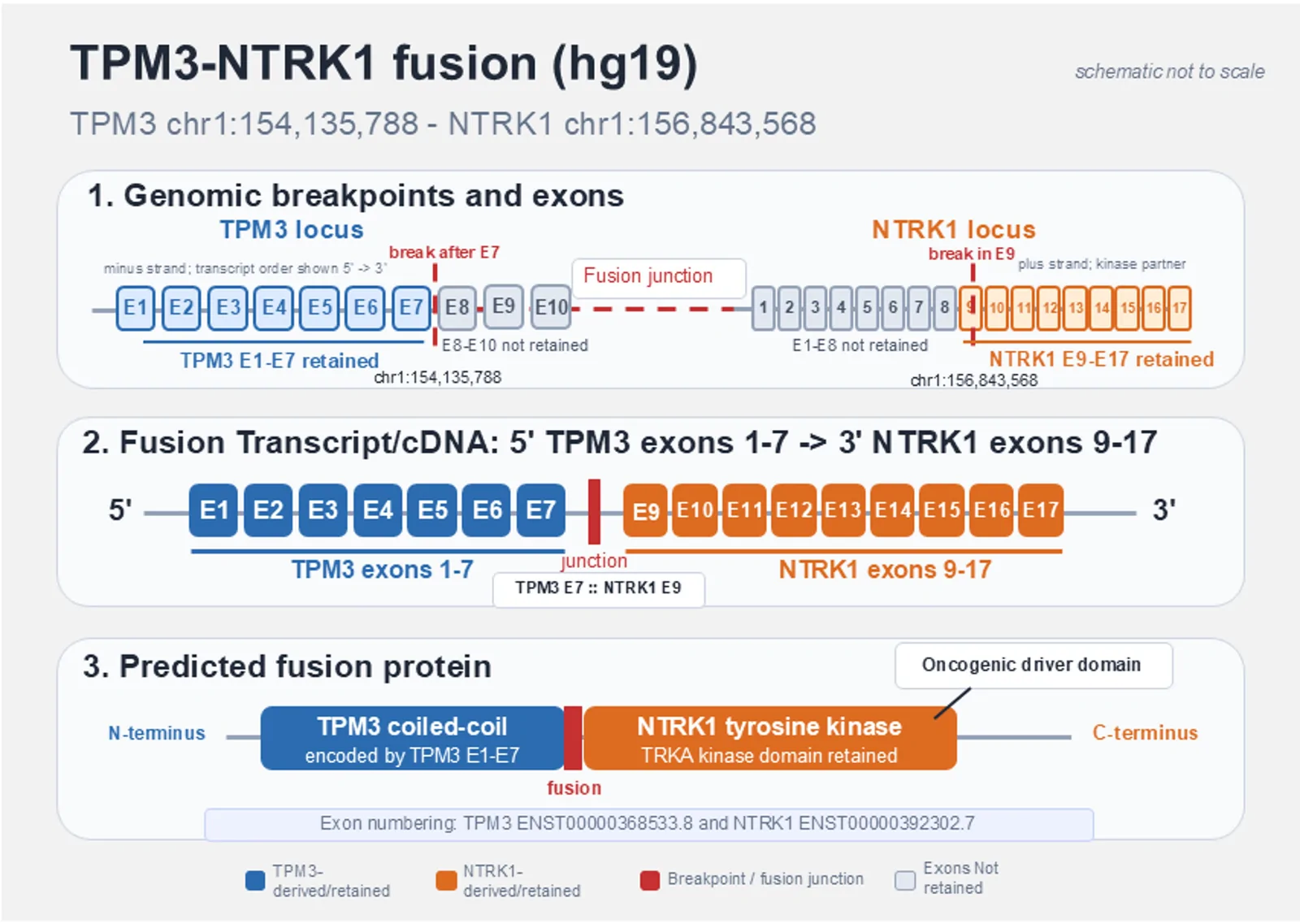
Schematic representation of the TPM3-NTRK1 gene fusion. The diagram represents an hg19-referenced intrachromosomal fusion joining the 5’/N-terminal portion of TPM3 to the 3’/C-terminal portion of NTRK1 at genomic coordinates TPM3 chr1:154,135,788 and NTRK1 chr1:156,843,568. Exon labels indicate the annotated fusion junction, TPM3 exon 7 fused to NTRK1 exon 9. The retained TPM3 segment contains the N-terminal coiled-coil/dimerization region, while the retained NTRK1 segment encodes the C-terminal TRK tyrosine kinase domain.

CT-guided biopsy of a progressing right lung nodule was analyzed by Guardant360 Tissue NGS (742-gene DNA + 367-gene RNA). TPM3::NTRK1 fusion was identified as the dominant alteration on both DNA (VAF 30.9%) and RNA (6,109 transcripts per million [TPM]), alongside residual KIF5B::RET (VAF 6.9%; RNA 2,942 TPM) (**Figure 1**). No secondary RET kinase domain mutations were detected. Co-occurring alterations included CDKN2A biallelic homozygous deletion, ATM, CHEK2, BRCA2, and RB1 loss, MTAP loss, and RAD51C promoter methylation. TMB was 7.97 mut/Mb; microsatellite status was stable; PD-L1 TPS was <1%.

A concurrent Guardant360 Liquid (cfDNA) assay detected no reportable somatic alterations; critically, neither KIF5B::RET nor TPM3::NTRK1 was captured, with tumor fraction <0.05%. This tissue-liquid discordance underscores the limitations of cfDNA-based assays for detecting fusion events at low tumor shedding and the necessity of tissue re-biopsy with RNA-inclusive NGS at progression (14).

**Figure 2:**
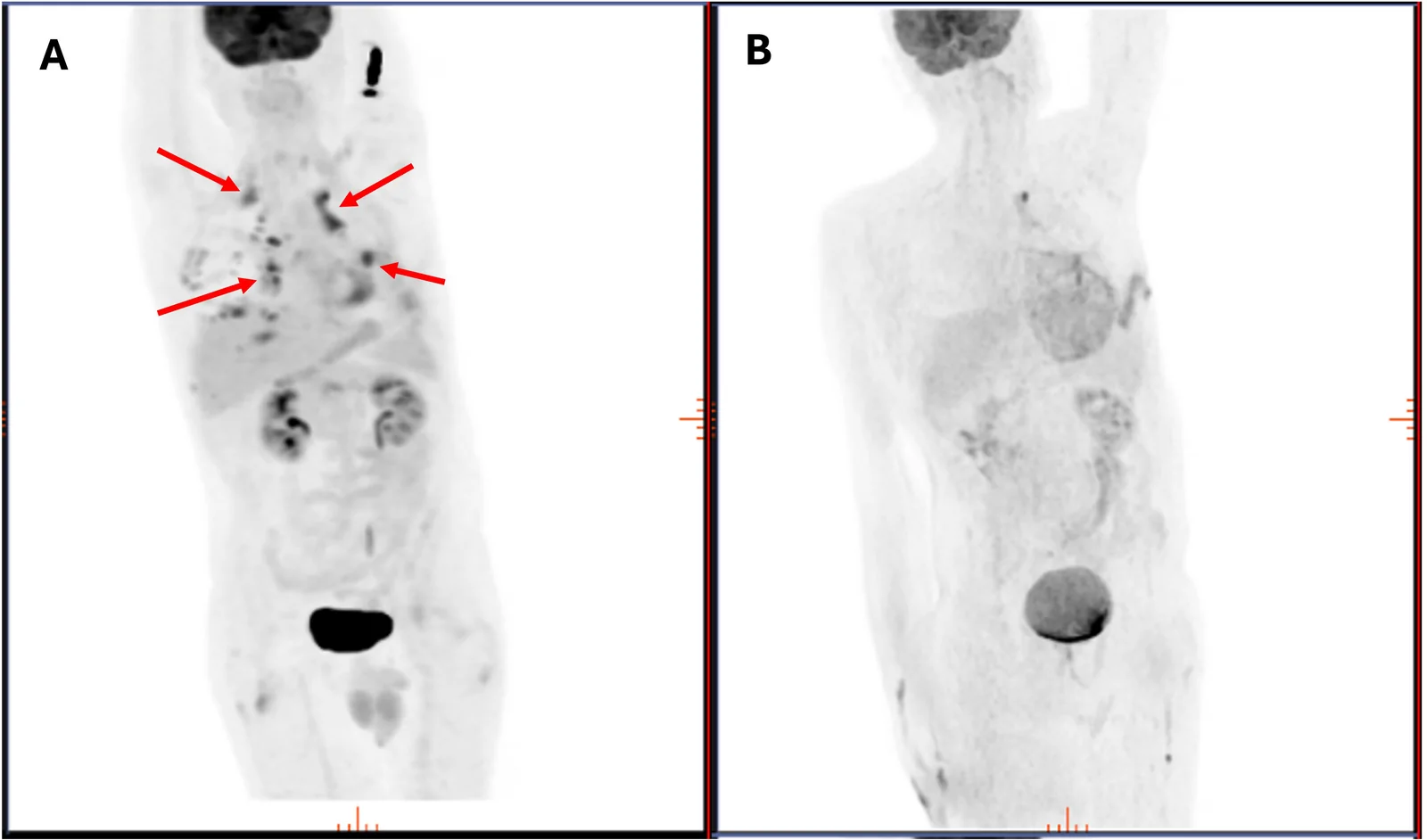
Whole-body 18F-FDG PET coronal images demonstrating metabolically active bilateral pleural thickening, bilateral pulmonary nodules, bilateral pleural effusions, skeletal lesions, and internal mammary lymphadenopathy in panel **(A)** (red arrows). Panel **(B)** shows interval regression of metabolic activity at most disease sites, consistent with a partial treatment response.

Selpercatinib (dose-escalated from 120 to 160 mg twice daily) and larotrectinib (100 mg twice daily) were commenced at Month 106. Baseline and follow-up ECG and echocardiography findings were normal. Treatment was well tolerated with no derangement in liver function tests (LFTs) and no evidence of cardiotoxicity. At eight weeks, FDG PET-CT demonstrated metabolic response with reduction in tumor burden and increasing consolidative change (**Figure 2**). These consolidative parenchymal changes were seen within the prior SBRT field and were consistent with post-radiation fibrosis. There was no increased metabolic activity in these areas and concordant metabolic regression at all non-irradiated sites confirming a responding disease. Concurrently, serum sodium improved from 103 to 128 mmol/L. No grade ≥3 treatment-related adverse events were observed.

## Discussion

RET fusions are approximately seen in 1-2% of non-small cell lung cancers (NSCLC). They carried a poor prognosis given the limited efficacy of chemotherapy and earlier multikinase agents until the advent of selective inhibitors (1) (2) like selpercatinib which is a potent and highly selective CNS-penetrant RET inhibitor. It has significantly improved clinical outcomes, demonstrating durable and sustained responses in the pivotal LIBRETTO-001 study (1) (3).

Acquired resistance to selpercatinib, however, remains a challenge, and the mechanisms underpinning it are still being defined. These are mediated by either secondary mutations affecting the RET kinase (on-target mechanisms) or compensatory activation of parallel signaling pathways that sustain tumor growth despite RET blockade (off-target mechanisms) (15).

We describe a patient with KIF5B::RET-positive lung adenocarcinoma who achieved a long term response to selpercatinib before progressing with acquisition of a TPM3::NTRK1 fusion (**Figure 1**) as an off-target bypass mechanism. The subsequent response (radiological and clinical response with resolution of cancer progression related to SIADH) to dual RET and TRK blockade provides the first clinical validation of this combination strategy in the NTRK1-resistance setting (**Figure 2**).

To the best of our knowledge, this is the first published case of NTRK1 fusion (rather than NTRK3) which emerged as a mechanism of resistance to selective RET inhibition. A major strength of this case was the use of tissue biopsy and liquid biopsy (DNA and RNA-based) NGS approaches at progression (**Figure 1**). The RNA sequencing plays a critical role in identifying potentially actionable fusion events that may be missed by DNA-based assays alone (14), as observed in this case. Another notable aspect of this case was the discordance between liquid and tissue-based genomic profiling. Both the RET and NTRK fusions were undetected on liquid biopsy but were identified on tissue-based NGS, underscoring the complementary roles of tissue and liquid biopsy and the importance of comprehensive genomic profiling using both modalities.

Secondary RET mutations at the solvent-front residue G810 are the most common on-target event and account for roughly 10% of resistance cases. They work by physically blocking drug binding at the kinase active site (5). Notably, several next-generation selective RET inhibitors including LOXO-260, EP0031, and SY-5007 are in clinical development with activity against G810 solvent-front mutations and improved CNS penetration (16).

Off-target mechanisms are more heterogeneous and typically involve reactivation of the MAPK or PI3K/AKT signaling axes through an alternative molecular route like MET amplification, mutations in RAS family genes, and alterations in BRAF and FGFR (5).

In our patient, no secondary RET mutation was found. The resistance biopsy showed instead was a TPM3::NTRK1 fusion at a variant allele fraction (VAF) of 30.9%, alongside the original KIF5B::RET fusion at a much lower VAF of 6.9% (**Figure 1**). That the NTRK1-bearing clone had overtaken the RET-driven population in abundance by the time of progression speaks directly to Darwinian selection as described by Subbiah et al. (6). This is the molecular equivalent of cutting off the dominant head of the Hydra, only to find another has grown in its place as documented across RET-driven tumors, BRAF-inhibited and EGFR-inhibited malignancies (6) (7).

Two prior cases have reported NTRK fusions arising as resistance to selpercatinib. In 2021, Subbiah et al. (8) described a patient with KIF5B::RET-positive lung cancer who progressed after approximately 10 months on selpercatinib. A KHDRBS1::NTRK3 fusion was subsequently identified on archived tissue, but only after the patient had died, rendering the finding scientifically important yet clinically moot (8). The 2024 case from the same group involved an ETV6::NTRK3 fusion in a patient with RET-mutant medullary thyroid carcinoma, where earlier detection allowed serial adaptive management (6).

The present case diverges from both in one fundamental respect: the NTRK gene involved is NTRK1, not NTRK3. NTRK1 encodes TrkA, while NTRK3 encodes TrkC. These are distinct receptor tyrosine kinases with overlapping but not identical downstream targets and differing sensitivities to some inhibitors (12). The fusion partner, TPM3, also has notable historical significance. The TPM3::NTRK1 rearrangement was the first oncogenic gene fusion identified in human cancer (originally described in colorectal carcinoma) by Martin-Zanca, Hughes, and Barbacid in 1986 (9).

Subsequently, Vaishnavi et al. demonstrated that TPM3::NTRK1 is an oncogenic and therapeutically actionable driver in lung adenocarcinoma. Similar to KIF5B::RET, it also activates MAPK and PI3K/AKT pathways (10) (11). This proves that the fusion is likely an acquired resistance mechanism rather than the primary driver mutation and this is supported by the broad genomic profiling report, which listed only driver alterations detected after progression on selpercatinib, including the NTRK1 fusion which was missing in the initial biopsy specimen.

Subbiah et al. (2021) (8) demonstrated in BaF3 cell models that selpercatinib alone was insufficient to suppress dual RET/NTRK3-positive populations and proposed the use of combined RET and TRK inhibition as a rational therapeutic strategy (8). This concept was subsequently translated into clinical practice in a reported case of medullary thyroid carcinoma in 2024 (6). Guided by these findings, our patient was commenced on dual targeted therapy with selpercatinib and larotrectinib, consistent with larotrectinib’s established pan-NTRK efficacy (ORR 79%; median duration of response >35 months in a pooled analysis) (12) (13), with good early tolerability and no significant adverse side effects.

Unlike the previously reported case (8), NTRK3 fusion was detected only retrospectively due to limited ctDNA assay coverage and therefore provided no clinical benefit (8). In contrast, our patient underwent tissue re-biopsy and comprehensive genomic profiling using Guardant360 Tissue and liquid biopsy at progression and the TPM3::NTRK1 fusion was confirmed on both DNA and RNA sequencing, with high RNA expression supporting its functional relevance.

RNA-based NGS is increasingly recognized as the preferred approach for detecting NTRK fusions, as it directly identifies expressed fusion transcripts and overcomes limitations of DNA-only assays (14). This highlights the use of tissue re-biopsy with both these methods at progression to avoid missing actionable resistance mechanisms (14).

The limitations of this report include its single-patient design, from which no generalizable conclusions can be drawn. The co-occurring genomic alterations at resistance (CDKN2A homozygous deletion, losses of ATM, CHEK2, BRCA2, and RB1, and MTAP deletion alongside RAD51C promoter methylation) reflect a tumor under considerable genomic stress, but their prognostic significance and potential therapeutic relevance in this specific context are still not clear and require further studies (4).

In conclusion, in this era of precision oncology, acquired resistance has evolved from being viewed as an exception to becoming an expected manifestation of tumor adaptation under sustained therapeutic pressure. This necessitates the recognition of these resistance-directed pathways and the need for performing comprehensive molecular profiling (both DNA and RNA based NGS panels) at progression to optimize subsequent treatment strategies including personalized combination therapies.

## Data Availability

The original contributions presented in the study are included in the article/**Supplementary Material**. Further inquiries can be directed to the corresponding author.
