# Supplementary material for: Acquired TPM3::NTRK1 fusion as a novel off-target resistance mechanism to selpercatinib in RET-positive lung adenocarcinoma: a case report
Source: Front Oncol. 2026 Aug 28;16:1936583. doi: 10.3389/fonc.2026.1936583 (PMC13561901; doi:10.3389/fonc.2026.1936583)
Supplement: Supplementary file 1 [file Table1.docx]

**CARE Checklist (2013)**

*of information to include when writing a case report*

**Article title:** Acquired TPM3::NTRK1 Fusion as a Novel Off-Target Resistance Mechanism to Selpercatinib in RET-Positive Lung Adenocarcinoma: A Case Report

| **Topic** | **Item** | **Checklist Item Description** | **Reported on Page** | **Line / Paragraph** |
| --- | --- | --- | --- | --- |
| Title | 1 | The words "case report" in the title along with the area of focus | Page 1 | Title |
| Keywords | 2 | 2 to 5 key words that identify areas covered in this case report | Page 1 | Keywords section: RET fusion, selpercatinib, acquired resistance, NTRK1 fusion, larotrectinib, lung adenocarcinoma, case report |
| Abstract | 3a | Introduction - What is unique about this case? What does it add to the medical literature? | Page 1 | Abstract paragraph -first 3 sentences |
|  | 3b | The main symptoms of the patient and the important clinical findings | Page 1 | Abstract - sentences 4-6 |
|  | 3c | The main diagnoses, therapeutic interventions, and outcomes | Page 1 | Abstract - sentences 7-10 |
|  | 3d | Conclusion - What is the main "take-away" lesson from this case? | Page 1 | Abstract - final 2 sentences |
| Introduction | 4 | Briefly summarizes why this case is unique with medical literature references | Page 2 | Introduction section - single paragraph with references [3][6][8] |
| Patient Information | 5a | De-identified patient demographic and other relevant information | Page 2 | Case Presentation para 1: "A male in his late 40s, a non-smoker..." |
|  | 5b | Main concerns and symptoms of the patient | Page 2 | Case Presentation para 3: dyspnoea, hyponatraemia, SIADH |
|  | 5c | Medical, family, and psychosocial history including relevant genetic information | Page 2 | Para 1: no smoking history; comorbidities (hypothyroidism, HTN) not detailed to protect anonymity; KIF5B::RET as genetic driver |
|  | 5d | Relevant past interventions with outcomes | Page 2 | Paras 1-2: lobectomy (pT1aN0), pemetrexed-cisplatin, maintenance (~54 cycles), nab-paclitaxel bridge, selpercatinib 46 months, SBRT x3 |
| Clinical Findings | 6 | Relevant physical examination and important clinical findings | Page 2 | Para 3: dyspnoea, severe hyponatraemia (Na⁺ 103), bilateral pleural effusions, SIADH biochemistry |
| Timeline | 7 | Historical and current information organized as a timeline (figure or table) | Table 1 | Table 1: Disease course and key interventions (Month 1 through Month 108) |
| Diagnostic Assessment | 8a | Diagnostic methods (PE, laboratory testing, imaging, surveys) | Pages 2-3 | Paras 4-5: CT-guided biopsy, Guardant360 Tissue NGS (742-gene DNA + 367-gene RNA), Guardant360 Liquid (cfDNA), PET-CT, serial MRI brain |
|  | 8b | Diagnostic challenges | Page 3 | Para 5: cfDNA assay failed to detect either fusion (tumour fraction <0.05%); tissue-liquid discordance. Reference [14] |
|  | 8c | Diagnosis (including other diagnoses considered) | Pages 2-3 | Lung adenocarcinoma with KIF5B::RET (primary driver); acquired TPM3::NTRK1 (resistance mechanism). SIADH (paraneoplastic). On-target RET mutations excluded. |
|  | 8d | Prognosis (such as staging in oncology) where applicable | Page 2 | Initial: pT1aN0. At resistance: TMB 7.97 mut/Mb, MSS, PD-L1 <1%, co-alterations (CDKN2A, ATM, CHEK2, BRCA2, RB1, MTAP loss) |
| Therapeutic Intervention | 9a | Types of intervention (pharmacologic, surgical, preventive, self-care) | Pages 2-3 | Surgical: lobectomy. Pharmacologic: pemetrexed-cisplatin, pemetrexed maintenance, nab-paclitaxel, selpercatinib, weekly paclitaxel (bridge), selpercatinib + larotrectinib. Radiotherapy: SBRT x3 |
|  | 9b | Administration of intervention (dosage, strength, duration) | Page 3 | Selpercatinib 120🡪160 mg BD; larotrectinib 100 mg BD. Duration: selpercatinib 46 months; combination therapy from Month 106.  SBRT doses: 35 Gy/5#, 45 Gy/3#, 35 Gy/5# |
|  | 9c | Changes in intervention (with rationale) | Pages 2-3 | Sequential changes documented: surgery 🡪 chemotherapy (recurrence) 🡪 maintenance (disease control) 🡪 nab-paclitaxel (bridge) 🡪 selpercatinib (RET driver) 🡪 SBRT x3 (oligoprogression) 🡪 weekly paclitaxel (bridge pending NGS) 🡪 dual targeted therapy (NTRK1 resistance identified) |
| Follow-up and Outcomes | 10a | Clinician and patient-assessed outcomes | Page 3 | Month 107: good tolerability, ambulatory, Na⁺ 116. Month 108: PET-CT metabolic response, lesion size reduced, consolidative change, Na⁺ 128, BiPAP requirement decreasing |
|  | 10b | Important follow-up diagnostic and other test results | Page 3 | 8-week PET-CT: metabolic response (Figure 1 Panel B). Serial sodium: 103 🡪 116 🡪 128 mmol/L. ECG and echocardiography normal. |
|  | 10c | Intervention adherence and tolerability (how was this assessed?) | Page 3 | Assessed by clinical review at 4 and 8 weeks. No dose modifications required. No derangement in LFTs. No cardiotoxicity. No grade ≥3 adverse events. |
|  | 10d | Adverse and unanticipated events | Page 3 | No treatment-related adverse events recorded. Hyponatraemia attributed to paraneoplastic SIADH (disease-related, not drug-related). IPC-related management described. |
| Discussion | 11a | Strengths and limitations in your approach to this case | Pages 3-5 | Strengths: first NTRK1 (vs NTRK3) resistance case; real-time identification enabling actionable intervention; tissue + liquid biopsy discordance documented; dual DNA/RNA confirmation. Limitations: single-case design; no serial molecular monitoring during selpercatinib; co-alterations of uncertain significance; early follow-up. |
|  | 11b | Discussion of the relevant medical literature | Pages 3-5 | References [1]-[15]: selpercatinib efficacy, RET resistance landscape, prior NTRK3 cases (Subbiah 2021/2024), Darwinian resistance framework, TPM3::NTRK1 biology (Martin-Zanca 1986; Vaishnavi 2013), larotrectinib efficacy, NTRK detection methodology |
|  | 11c | The rationale for conclusions (including assessments of cause and effect) | Pages 4-5 | VAF inversion (NTRK1 30.9% > RET 6.9%) supports Darwinian clonal selection. Absence at initial profiling and emergence only at resistance confirms acquired mechanism. Response to dual inhibition validates preclinical BaF3 modelling [8]. |
| Patient Perspective | 12 | The patient should share their perspective or experience whenever possible | N/A | Not obtained. Patient consented to publication but a formal patient perspective statement was not collected. |
| Informed Consent | 13 | The patient should give informed consent | Ethics Statement | Written informed consent obtained from the patient for publication. Statement included in the Ethics Statement section. |
